# MARTs and MARylation in the Cytosol: Biological Functions, Mechanisms of Action, and Therapeutic Potential

**DOI:** 10.3390/cells10020313

**Published:** 2021-02-03

**Authors:** Sridevi Challa, MiKayla S. Stokes, W. Lee Kraus

**Affiliations:** 1 Laboratory of Signaling and Gene Regulation, Cecil H. and Ida Green Center for Reproductive Biology Sciences, University of Texas Southwestern Medical Center, Dallas, TX 75390, USA; mikayla.stokes@utsouthwestern.edu; 2 Division of Basic Research, Department of Obstetrics and Gynecology, University of Texas Southwestern Medical Center, Dallas, TX 75390, USA; 3 Program in Genetics, Development, and Disease, Graduate School of Biomedical Sciences, University of Texas Southwestern Medical Center, Dallas, TX 75390, USA

**Keywords:** ADP-ribose (ADPR), ADP-ribosylhydrolase, ADP-ribosylation (ADPRylation), mono(ADP-ribosylation) (MARylation), poly(ADP-ribosylation) (PARylation), mono(ADP-ribosyl) transferase (MART), poly(ADP-ribosyl) polymerase (PARP), nicotinamide adenine dinucleotide (NAD^+^), PARP inhibitor, mass spectrometry, chemical genetics

## Abstract

Mono(ADP-ribosyl)ation (MARylation) is a regulatory post-translational modification of proteins that controls their functions through a variety of mechanisms. MARylation is catalyzed by mono(ADP-ribosyl) transferase (MART) enzymes, a subclass of the poly(ADP-ribosyl) polymerase (PARP) family of enzymes. Although the role of PARPs and poly(ADP-ribosyl)ation (PARylation) in cellular pathways, such as DNA repair and transcription, is well studied, the role of MARylation and MARTs (i.e., the PARP ‘monoenzymes’) are not well understood. Moreover, compared to PARPs, the development of MART-targeted therapeutics is in its infancy. Recent studies are beginning to shed light on the structural features, catalytic targets, and biological functions of MARTs. The development of new technologies to study MARTs have uncovered essential roles for these enzymes in the regulation of cellular processes, such as RNA metabolism, cellular transport, focal adhesion, and stress responses. These insights have increased our understanding of the biological functions of MARTs in cancers, neuronal development, and immune responses. Furthermore, several novel inhibitors of MARTs have been developed and are nearing clinical utility. In this review, we summarize the biological functions and molecular mechanisms of MARTs and MARylation, as well as recent advances in technology that have enabled detection and inhibition of their activity. We emphasize PARP-7, which is at the forefront of the MART subfamily with respect to understanding its biological roles and the development of therapeutically useful inhibitors. Collectively, the available studies reveal a growing understanding of the biochemistry, chemical biology, physiology, and pathology of MARTs.

## 1. Introduction

### 1.1. ADP-ribosylation and the PARP Family

**Figure 1:**
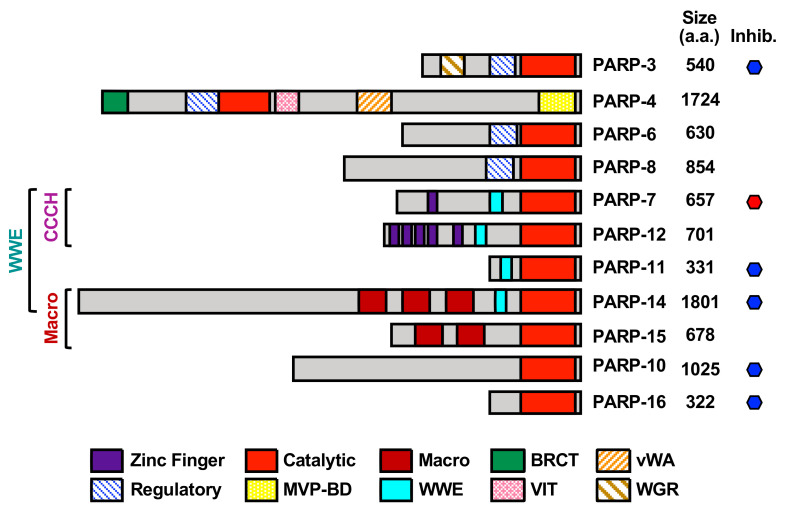
Structural and functional organization of mono(ADP-ribosyl) transferases (MARTs). Schematic representations of human MARTs depicting key structural and functional domains. The MARTs have been categorized based on the presence of CCCH zinc fingers, tryptophan-glutamate (WWE) motifs, and macrodomains in the proteins. The domains shown are MVP-BD (major vault protein-binding domain); BRCT (BRCA1 C-terminal domain); VIT (vault inter-trypsin domain); RRM (RNA Recognition Motif); WGR (tryptophan-glycine-arginine-rich domain); and vWA (von Willebrand factor type A domain). The MARTs that have known inhibitors (inhib.) are indicated with a hexagon (red = in clinical trials, blue not yet in clinical trials). Other non-cytosolic or non-MART members of the poly(ADP-ribosyl) polymerase (PARP) family are not shown: PARP-1, PARP-2, and PARP5a/b (nuclear polyenzymes); PARP-9 and PARP-13 (catalytically inactive). PARP-3 is a MART, but is primarily nuclear. The size of each protein (in amino acids, a.a.) is drawn to scale, but the position and size of the functional domains is approximate.

ADP-ribosylation (ADPRylation) is a regulatory post-translational modification (PTM) of proteins on a range of amino acid residues (including Asp, Glu, Ser, Cys, Lys, Arg) that results in the reversible attachment of ADP-ribose (ADPR) subunits on substrate proteins, acting to control their functions through a variety of mechanisms. Members of the poly(ADP-ribosyl) polymerase (PARP) family of enzymes play a key role in catalyzing cellular ADPRylation. The mammalian PARP family contains 17 members, each possessing an ADP-ribosyltransferase catalytic domain that is functionalized with other domains that confer additional biochemical functions or direct the proteins to specific cellular compartments [1,2] (Table 1, Figure 1). While mono(ADP-ribosyl) transferases (MARTs) (PARP “monoenzymes”) modify their target proteins by the addition of a single ADPR moiety (i.e., mono(ADP-ribosyl)ation (MARylation)), PARP “polyenzymes” catalyze the formation of branched or linear chains of multiple ADPR moieties (i.e., poly(ADP-ribosyl)ation (PARylation)) [3,4,5].

### 1.2. Determinants of PARP Catalytic Activity and Substrate Specificity

Specificity for PARylation is conferred, in part, by the presence of a histidine-tyrosine-glutamate (H-Y-E) sequence triad motif in the active site of the PARP polyenzymes. The glutamate residue in the H-Y-E triad is required for the elongation of the PAR chain, and this sequence is altered in PARP monoenzymes [6] (Table 1). PARP-3, however, contains an H-Y-E motif, but functions as a MART [6]. The catalytically active MARTs contain variations of this sequence in which the glutamate residue is replaced with leucine (L; PARP-14), isoleucine (I, PARPs 6, 7, 8, 10, 11, 12), or tyrosine (PARP-16) (Table 1).

In addition to differences in the catalytic triad, the catalytic activity of PARP family members is also determined by additional functional domains within the proteins, as well as interactions with other macromolecules (e.g., proteins, DNA, RNA). For example, the helical subdomain (HD) of the catalytic domain in PARP-1 functions as an autoinhibitory domain that blocks productive nicotinamide adenine dinucleotide (NAD^+^) binding until released [7]. Other domains that drive macromolecular interactions may also impact the catalytic activity of PARP family members. For example, the tryptophan-glycine–arginine-rich (WGR) domain found in DNA-dependent PARP polyenzymes (i.e., PARP-1, 2 and 3) may bind to damaged DNA; the CCCH zinc fingers in PARP-7, 12, and 13 bind to RNA; the RNA recognition motifs (RRMs) in PARP-10 and PARP-14 bind RNA, and the tryptophan-glutamate (WWE) domains in PARP-7, 12, 13, 14 may bind to PAR chains (Figure 1) [8,9]. Recent studies have also shed light on the role of accessory proteins that direct the substrate specificity of PARPs. For example, histone PARylation factor 1 (HPF1) directs PARP-1 catalytic activity to Ser residues in histone proteins when cells undergo DNA damage [10,11], and NMNAT-1 directs PARP-1 activity towards Asp/Glu residues in histones when PARP-1 is activated by physiological activators, such as snoRNAs [12,13]. Of note, although the recombinant catalytic domain of PARP-4 has MARylation activity, PARP-4 has the ability to synthesis PAR chains when localized to vault particles [14]. Additional studies are required to understand the regulators of PARP-4 catalytic activity and other poorly studied PARPs [15,16].

### 1.3. ADP-ribosylhydrolases and the Reversal of ADPRylation

ADP-ribosylation is a reversible post-translational modification (PTM) that can be removed by a set of hydrolases (i.e., ‘erasers’) that have distinct specificities for MAR and PAR: PAR glycohydrolase (PARG) hydrolyzes PAR chains, whereas MacroD1, MacroD2, and ADP-ribosylhydrolase 1 (ARH1) remove MAR. Other ADP-ribosylhydrolases, such as ARH3, TARG1, NuDT9, NuDT16, and ENPP1 act on both MAR and PAR [17,18]. The activity of some ADP-ribosylhydrolases are specific to the residues of modification. For example, ARH1 acts on MARylated arginine, ARH3 acts on MARylated serine, and MacroD1/2 act on MARylated aspartate and glutamate [17,18]. Expression of these ADP-ribosylhydrolases in cells directs the levels of cellular PAR and MAR. In this regard, the expression of PARG results in higher levels of MARylation in the nuclei of cells subjected to DNA damage and loss of MacroD2 leads to accumulation of MARylated PARP-1 [19,20].

Macrodomains are a class of protein domains that act as ‘readers’ of ADP-ribosylation due to their ability to bind to ADPR. Some macrodomains possess intrinsic ADP-ribosylhydrolase activity and this can act as ADPR erasers as well. The nsP3 protein found in coronaviruses contains macrodomains that can bind and hydrolyze ADPR [21]. Recent studies have uncovered the importance of the ADPR binding and hydrolase activities of these macrodomains on virulence [21].

### 1.4. MARTs and Cytosolic MARylation

Of the 17 PARP family members, the majority are cytosolic MARTs (PARPs 4, 6, 7, 8, 10, 11, 12, 14, 15, and 16) (Table 1). The MARTs have distinct structural and functional domains, subcellular localizations, and affinities for their shared substrate, NAD^+^, which underlie their divergent functions (Figure 1) [1,2,6,22,23]. The MARTs are also capable of modifying a variety of amino acid residues, such as Ser, Asp, Glu, and Cys, thus diversifying the repertoire of MARylation targets [3,22]. These unique features of the MARTs also provide opportunities to generate research tools for specific detection of substrates and inhibition of their catalytic activities. In this review, we discuss recent advances in technologies to detect MARylation and prospects for inhibition of MARTs with small molecule drugs. In addition, we discuss the important cytosolic pathways regulated by MARTs, their mechanisms of actions, and their biological roles in the pathogenesis of disease.

## 2. New Strategies to Detect MARylation

The scope of research into MARylation has been limited by the inability to specifically detect MARylation and distinguish it from PARylation. In this section, we will discuss recent advances in technologies to detect MARylation.

### 2.1. Labeled Metabolites to Detect MARylation

**Figure 2:**
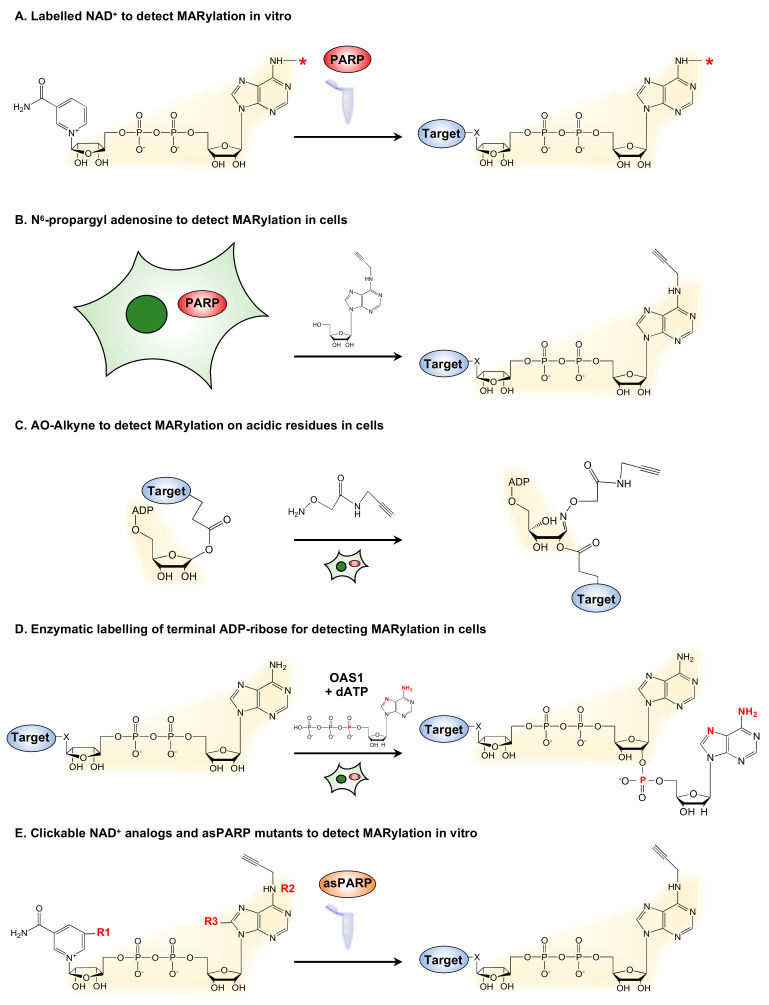
Strategies for the detection of mono(ADP-ribosyl)ation (MARylation). (**A**) Detection of in vitro MARylation using labeled nicotinamide adenine dinucleotide (NAD^+^). PARPs utilize fluorescently or radioactively labeled NAD^+^ to modify protein substrates that can then be detected by incorporation of the label. The asterisk (*) indicates a common site used for labelling. (**B**) N^6^-propargyl adenosine (N^6^pA) can be used to detect MARylation in cells. Metabolic labelling of cells with N^6^pA, followed by an azide-alkyne cycloaddition reaction, enables detection of MARylated proteins. (**C**) AO-Alkyne can be used to detect MARylation on acidic residues in cells. Treatment of cells with clickable AO-alkyne enables detection of MARylated proteins in cells. (**D**) Enzymatic labelling of terminal ADP-ribose (ELTA) enables detection of MARylation in vitro. Moreover, 2′-5′ oligoadenylate synthetase 1 (OAS1) is used to label free or protein-conjugated ADP-ribose with analogs of dATP. Highlighted in red are the residues available for various labels, such as fluorescent dyes and radioisotopes (e.g., ^32^P). (**E**) Analogs of NAD^+^ can be used to identify targets of specific MARTs with an asPARP approach. Engineered mutations in MARTs ensure specific utilization of the NAD^+^ analogs to modify the target proteins. The NAD^+^ analogs may be modified as follows: (1) simultaneously on the nicotinamide moiety (R1) to confer analog sensitivity and at position 6 of adenine (R2) to add a clickable moiety (alkyne shown; or azide) [30,31] or (2) at position 8 of adenine, both to confer analog sensitivity and to add a clickable moiety (alkyne or azide; not shown) [32,33]. By using clickable NAD^+^ analogs, substrates can be identified by labeling with fluorescent dyes or biotin, or by mass spectrometry. The clickable residues are highlighted.

Since PARPs utilize NAD^+^ as a substrate and a source of ADPR, historic studies of (ADP-ribosyl)ation (ADPRylation) have used radioactively- or biotin-labelled NAD^+^ to visualize target modification (Figure 2A). By coupling labelled NAD^+^ with in vitro assays, specific protein substrates of PARPs can be identified. Similarly, by coupling biotin-labeled NAD^+^ and protein microarrays, Feijs et al. (2013) have identified several MARylation targets of PARP-10 and PARP-14 [24]. The use of labelled NAD^+^ for detecting MARylation is limited to in vitro assays, as NAD^+^ is not cell membrane-permeable and requires manipulations to permeabilize the membrane [25]. Hence, other approaches for generating labelled ADP-ribose are required to identify physiological MARylation targets.

One such technique uses N^6^-propargyl adenosine (N^6^pA), which can be used in intact mammalian cells, followed by click chemistry to generate fluorescently labeled ADPR on target proteins (Figure 2B) [26]. Another approach is the use of a clickable aminooxy alkyne (AO-alkyne) probe that recognizes the free aldehyde on MARylated acidic residues, such as aspartate and glutamate. By performing click reaction in cellular lysates, this probe enables detection of MARylated substrates that were modified in vivo or intact cells (Figure 2C) [27,28]. Another recently developed method, called enzymatic labeling of terminal ADP-ribose (ELTA), uses the human enzyme 2′−5′ oligoadenylate synthetase 1 (OAS1) to tag the 2′-OH of the terminal ADPR with analogs of dATP that are amenable to tagging with fluorescent or affinity tags (Figure 2D) [29]. Collectively, these metabolite labeling techniques provide a suite of techniques for detecting ADPRylation, although not always with specificity for MARylation.

### 2.2. Antibodies to Detect MARylation

MARylation of the diphthamide 715 residue in eEF2 is the earliest known MARylation modification [34]. MARylation of eEF2 by bacterial toxins, such as diphtheria toxin, leads to a complete shutdown of host mRNA translation and enhanced virulence [35,36]. Although earlier efforts to identify MAR-specific antibodies for eEF2 were not very successful, Siegmund et al. (1992) successfully generated specific rabbit polyclonal antibodies to MARylated *S. acidocaldarius* eEF2 by cross-adsorbing the non-specific antibodies with *S. acidocaldarius* eEF2 expressed in and purified from *E. coli* that do not contain the diphthamide residue [37]. This antibody was used in Western blotting and ELISA assays, but the full repertoire of applications for this antibody is not known.

**Figure 3:**
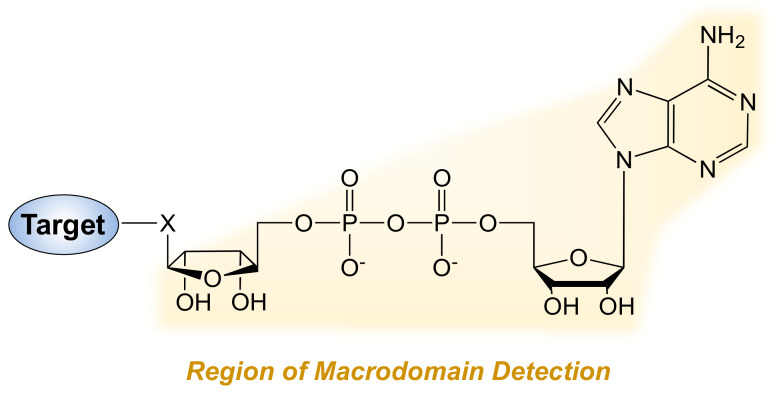
Detection of ADP-ribose (ADPR) by macrodomains. The chemical structure of a MARylated residue with the region of ADPR that is detected by PARP-14 (and other) macrodomains highlighted. Other ADPR binding motifs/domains (e.g., WWE motifs and PBZ domains) recognize distinct regions of the ADPR. Macrodomains 2 and 3 in PARP-14 likely contact the MARylated amino acid residue or other amino acids in the substrate protein, explaining why the PARP-14 macrodomains do not efficiently bind the terminal (protein distal) ADPR moieties in PAR chains [41].

The field of MARylation has lagged behind in the development of MAR antibodies, but this shortcoming has been overcome by the use of naturally occurring MAR binding domains. A variety of proteins, including but not limited to ADPR hydrolases, have structural and functional domains (e.g., macrodomains, WWE domains, PAR-binding zinc fingers) that bind to specific forms of ADPR, including MAR [38,39,40]. Collectively, these domains provide full coverage for recognizing and distinguishing different forms of ADPR, such as MAR, PAR, and oligo(ADP-ribosyl)ation [41]. PARP-14 contains three macrodomains, with macrodomains 2 and 3 binding specifically to MAR (Figure 3). Immunoprecipitation with His-tagged human PARP-14 macrodomains detected MARylated substrates of PARP-10 [42]. Similarly, the macrodomain from human PARP-14 conjugated to GFP detected the levels of MARylation in *Drosophila* cells [43]. This approach identified a key role of PARP-16 in the regulation of Sec body formation after amino acid starvation in *Drosophila* [43].

Our lab has developed antibody-like MAR detection reagents by conjugating the macrodomains from human PARP-14 to the Fc region of rabbit IgG. This reagent, by virtue of the Fc region, has broad utility in immune-based assays, such as Western blotting, immunoprecipitation, and immunofluorescent staining [41]. By using a set of these antibody-like ADPR detection reagents with different ADPR binding domains to detect various types of ADPR, such as the WWE domain from human RNF146 (recognizes PAR) and the macrodomain from *A. fulgidus* AF1521 (recognizes both PAR and MAR), we previously identified significant correlations between the levels of ADPRylation, gene expression, sensitivity to PARP inhibitors, and clinical outcomes in ovarian cancer patients [44]. Similar studies can be expanded to identify the clinical relevance of MARylation using the PARP-14 macrodomains. The development of these ADPR-binding reagents has provided new opportunities to distinguish PARylation from MARylation.

Recent developments in chemical and enzymatic approaches to obtain ADPRylated peptides are advancing our ability to generate site-specific ADPR antibodies. Using PARP-1/ HPF1 complex to ADPRylate synthetic peptides on specific serine residues [10,11], Bonfiglio et al. (2020) have developed antibodies to detect site-specific histone serine ADPRylation [20]. In this strategy, the authors generated in vitro-PARylated histone peptides using the HPF1/PARP-1 complex, followed by enzymatic digestion using PARG to cleave the PAR chain to leave behind a single ADPR moiety. They then enriched for MARylated peptides using boronate affinity chromatography. Using a phage display-recombinant antibody technique, the authors were able to generate antibodies that detect either site-specific or pan-MARylation [20]. Since this technique utilizes PARP-1-mediated peptide ADPRylation to generate site-specific antibodies, antibodies that detect site-specific MARylation catalyzed by MARTs are not yet available. Recent success in generating site-specific glutamate ADPRylated histone peptides using a PARP-1/NMNAT-1 approach [12] suggests that a wider array of residues can be targeted using these methods. Several recent studies have described techniques for the synthetic chemical synthesis of MARylated peptides, but the yield of modified peptides using these techniques is not sufficient for generation of antibodies [45,46,47]. Hence, it is essential to improve the yields of chemical synthesis of MARylated peptides to enable the generation of MARylation site-specific antibodies.

### 2.3. Chemical Genetics and Mass Spectrometry Approaches to Detect MARylation

Several studies have used the ADPR-binding ability of the AF1521 macrodomain to identify MARylated proteins using mass spectrometric techniques. In these studies, the ADPRylated proteome was enriched by affinity purification using the macrodomain, followed by mass spectrometric analysis [48,49,50]. A variety of mass spectrometry methods have been applied to the study of ADPRylation, and variations and improvements are continually being developed; a detailed discussion is beyond the scope of this review, but can be found elsewhere [51,52,53,54]. Moreover, sequence modifications to the AF1521 macrodomain have increased its affinity for ADPR and may enhance its utility in these protocols [55]. One caveat with this approach is that the AF1521 macrodomain binds to both PAR and MAR and, hence, cannot distinguish between the MARylated and PARylated proteomes with high confidence [53]. This limitation may be overcome by using (1) PARP-14 macrodomains or pan-MARylation antibodies to enrich for the MARylated proteome [20,42] or (2) using chemical genetics approaches that connect specific PARPs to specific ADPRylation events [30,32].

Advances using chemical genetics have yielded NAD^+^ analog-sensitive PARP (asPARP) approaches that can detect PARP-specific ADPRylation events when coupled with mass spectrometry [30,32]. In the asPARP approach, the catalytic domain of a PARP or MART of interest is mutated to yield a protein that can uniquely bind and perform catalysis with a clickable unnatural NAD^+^ analog. The substrates and sites of modification labeled by the asPARP plus the NAD^+^ analog can be identified using mass spectrometry. Carter-O’Connell et al. (2014) have engineered the human PARP-10 and PARP-11 by mutating key residues in the substrate binding site of these PARPs to allow specific utilization of an NAD^+^ analog (Figure 2E) [30].

They identified novel targets of PARP-10 and PARP-11 that belong to important cellular pathways, such as ubiquitylation, autophagy and organization of nuclear pores [30]. Likewise, we have used a similar approach for human PARP-3 to identify a broad array of PARP-3 substrates, as well as the glutamate and aspartate sites on which they are modified [32]. This allowed us to identify key substrates in DNA repair, RNA processing, chromatin regulation, and transcription [32].

Recently, the Cohen and Kraus labs have independently applied their complementary asPARP approaches to PARP-7 [31,33]. In their analysis, Rodriguez et al. (2021) found that the inactive PARP family member, PARP-13, which plays a key role in regulating the antiviral innate immune response, is a major substrate of PARP-7 [31]. We observed that the PARP-7 ADPRylated proteome in ovarian cancer cells is enriched for cell-cell adhesion and cytoskeletal proteins. Specifically, we found that PARP-7 MARylates α-tubulin to promote microtubule instability, which may regulate ovarian cancer cell growth and motility [33]. Together, these studies revealed that cysteine and acid residues (glutamate and aspartate) are MARylation acceptors for PARP-7 and provided new insights into PARP-7 biology by identifying substrates [31,33]. One caveat of the asPARP approach is the inability of NAD^+^ to pass through the cell membrane. Hence, identification of targets using the asPARP approach is restricted to in vitro assays. Nevertheless, these technologies have enabled the identification of novel and essential roles of MARTs in the regulation of key cellular pathways, as described below.

## 3. Cytosolic Processes Regulated by MARylation and MARTs

Work by Vyas et al. (2013, 2014) has been instrumental in characterizing the activity of MARTs, as well as their functions based on cellular localization [2,6]. Many MARTs localize to the cytosol, with some having organelle-specific localization. Others have observed that PARP-7 and PARP-10 have dynamic subcellular localizations, shuttling between the nucleus and the cytosol [56,57]. An examination of cellular phenotypes upon siRNA-mediated knockdown of MARTs led to some surprising observations [2]. Depletion of PARP-7 resulted in mitotic spindle defects, but did not affect cell viability. Depletion of PARP-14 resulted in decreased cell viability, as well as cytoskeleton defects. Depletion of PARP-4, PARP-6, and PARP-10, however, did not affect cell viability or promote morphological defects. Depletion of PARP-16 resulted in a morphological phenotype of round cells found in pairs, whereas depletion of PARP-8 resulted in round individual cells together with the most striking decrease in cell viability [2]. Together, these studies revealed the vast potential for MART enzymes in cellular processes. Below, we highlight selected examples.

### 3.1. PARP-16 and the Unfolded Protein Response

**Figure 4:**
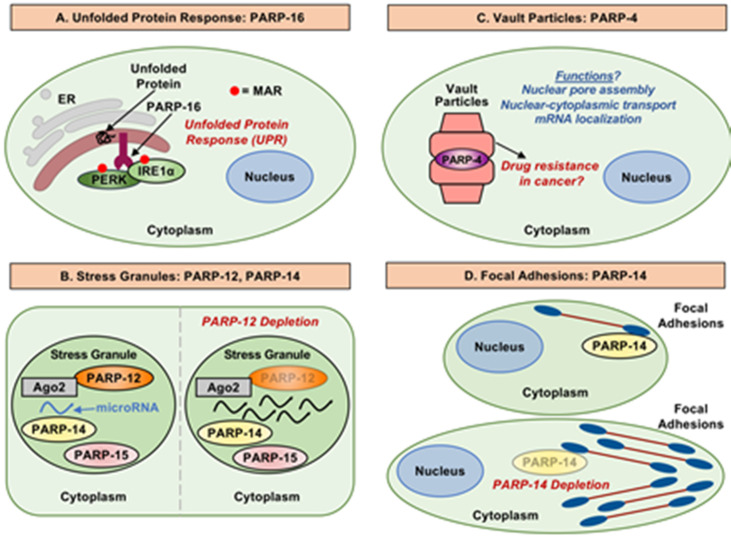
Biological processes regulated by cytosolic MARTs. (**A**) PARP-16 regulates the unfolded protein response (UPR) by modulating the activity of PERK and IRE1α through MARylation. (**B**) PARP-12, PARP-14, and PARP-15 are components of stress granules. PARP-12 associates with Ago2; upon PARP-12 depletion, microRNA levels increase. (**C**) PARP-4 is a component of vault particles, which are thought to play important roles in drug resistance in cancers. (**D**) PARP-14 is a component of focal adhesion complexes and is required for proper focal adhesion function and disassembly, as well as cell motility.

Jwa and Chang (2012) uncovered a role for PARP-16 and MARylation in the unfolded protein response (UPR) [58]. They found that PARP-16 localizes to the endoplasmic reticulum (ER) by virtue of its carboxyl-terminal transmembrane domain, which functions to anchor PARP-16 to the ER with the amino-terminal catalytic domain facing the cytoplasm. PARP-16 protein, as well as its catalytic activity, regulate the UPR signaling pathway and are required for ER stress responses [58]. In additional analyses, Jwa and Chang observed that PARP-16 activates two of the three main ER stress sensors—PERK and IRE1α; the third sensor, ATF6, was not regulated by PARP-16 [58]. Upon activation, PARP-16 MARylates PERK and IRE1α, and activates them by increasing their kinase activities, as well as IRE1α endonuclease activity (Figure 4A), key facets of the UPR [59]. Interestingly, the PARP-16 carboxyl-terminal luminal tail is also required for its function during ER stress, although the reasons are not clear [58]. This study highlights a key cytoplasmic functions of MARTs and MARylation, which are distinct from the historical focus on PARPs and PARylation in nuclear processes.

### 3.2. MARTs, Stress Granules, and mRNA

In an effort to explore potential roles for PAR in the regulation of mRNA, Leung et al. (2011) examined cytosolic stress granules [60]. Multiple MARTs were identified as components of heat shock-induced stress granules, including PARP-12, PARP-14, and PARP-15 (Figure 4B). Cytosolic stress granules are formed as a way to preserve and regulate mRNA translation and stability during cell stress [61]. Argonaute proteins 1–4, which bind small noncoding RNAs and are well known components of stress granules, are ADPRylated upon heat shock [62]. The accumulation of PAR in the stress granules was found to be required for regulation of microRNA mediated mRNA cleavage, as well as microRNA-mediated translational repression [60]. Although the link between PAR and the MARTs observed in this study is unclear, the results implicate cytosolic MARTs and MARylation in the post-transcriptional regulation of gene expression in stress granules. Interestingly, some MARTs contain ADPR-binding domains (i.e., WWE domains in PARPs 7, 11, 12, and 14; macrodomains in PARPs 9, 14, and 15) (Figure 1), which have the potential to functionally link MARTs to PARPs through PAR binding.

### 3.3. MARTs, RNA Binding, and RNA Processing

Catalytically active cytosolic MARTs (i.e., PARP-7, PARP-10, PARP-12, and PARP-14) are predicted to bind RNA through their RNA-binding ‘CCCH’ zinc fingers or RNA recognition motifs (RRMs) [8]. With the exception of PARP-10, these predicted RNA-binding PARPs also possess PAR binding domains (i.e., WWE domains or macrodomains) as noted above (Figure 1), providing a potential link between PAR binding, RNA binding, and RNA processing. PARP-14, acting in the cytoplasm, regulates mRNA stability. PARP-14 binds the RNA binding protein tristetraprolin (a.k.a. zinc finger protein 36 homolog or ZFP36) to promote the selective posttranscriptional control of macrophage tissue factor expression. The PARP-14/tristetraprolin complex binds an adenylate-uridylate-rich element (AU-rich element or ARE) in the 3′-UTR of tissue factor mRNA, promoting its degradation [63]. This is one of possibly many examples of RNA binding by cytosolic MARTs, which could regulate mRNA processing.

### 3.4. PARP-4 and Vault Particles

Vault particles are ribonucleoprotein particles composed of untranslated vault RNA molecules, along with major vault protein (MVP), PARP-4, and telomerase associated protein 1 (TEP-1) [14]. Although our understanding of the function of vault particles is rudimentary, vault particles have been implicated in cell transport, cell signaling, immune responses, and multidrug resistance (Figure 4C) [64,65,66,67]. Beyond being a component of vault particles, the role of PARP-4 is largely unknown. The uniqueness of PARP-4 protein is demonstrated by the domain organization (Figure 1). Along with being capable of MARylating substrate proteins, PARP-4 has also been shown to be capable of PARylating target proteins [15,16]. Increased PARP-4 expression in some drug resistant cell lines has led to speculation that PARP-4 is involved in drug resistance in cancer, possibly through its role in vault particles [65,68]. This idea is supported by an increased risk of carcinogen-induced colon tumor formation in *Parp4* knockout mice [69].

### 3.5. MARTs, MARylation, and the Cytoskeleton

The cytoskeleton of a cell, which comprises microtubules, actin filaments, and intermediate filaments, serves several key functions in the cell: it gives the cell its shape, organizes organelles, and provide a basis for movement, vesicle and organelle transport, as well as cell division [70]. A growing number of studies have connected MARTs to cytoskeleton proteins and the various functions of the cytoskeleton. We highlight a few examples below.

PARP-14 was identified by Vyas et al. (2013) as a participant in actin cytoskeleton regulation when they found a striking phenotype upon PARP-14 depletion [2]. In HeLa cells, siRNA-mediated knockdown of PARP-14 resulted in elongated cells with large dendritic-like protrusions [2]. The cells were unable to retract these protrusions efficiently. Subsequent work connected PARP-14 to focal adhesions (FAs)—large, dynamic, integrin-containing protein complexes through which the cytoskeleton of a cell connects to the extracellular matrix [71]. FAs purified from HFF-1 fibroblasts contain PARP-14 [72]. FA turnover (i.e., the process of breaking down FAs) is vital to the retraction of membrane protrusions [73]. PARP-14 depletion increases the strength of interaction between FAs and substrate, which may reduce FA disassembly (Figure 4D) [73]. Possible distinct roles for the catalytic activity and macrodomains of PARP-14 were not explored.

Likewise, PARP-7 has been shown to play roles in the regulation of microtubules and the mitotic spindle [2,33]. Microtubules, which form from polymers of α- and β-tubulin, are a part of the cytoskeleton, providing structure and shape to eukaryotic cells [74]. Microtubules form the major structural component of the mitotic spindle, a bipolar structure that segregates the chromosomes in mitosis [75]. siRNA-mediated knockdown of PARP-7 results in mitotic spindle defects [2]. In addition, we observed that PARP-7 MARylates α-tubulin to promote microtubule instability, which may regulate ovarian cancer cell growth and motility [33]. This effect is manifested as a PARP-7-dependent reduction in cellular microtubule content following recovery from depolymerization caused by cold or nocodozole. These examples with PARP-14 and PARP-7 highlight additional cytoplasmic functions of MARTs and MARylation that are very distinct from the historical focus on PARPs and PARylation in nuclear processes.

## 4. Roles for Cytosolic MARTs and MARylation in Health and Disease

The recent expansion in our knowledge of the cellular functions of MARTs and MARylation has been crucial to our understanding of their roles in health and disease. These advances have benefitted from substrate identification, genetic models, and gene expression analyses, capitalizing on the high expression levels of some MARTs in specific tissues or specific diseases. We review a few of the many examples of MARTs and MARylation in health and disease below.

### 4.1. PARP-6 and PARP-7 in Neuronal Development

Huang et al. (2016) sought to determine if MARTs play a physiological role in neurons, an idea suggested by high levels of MARylation in the brain [76]. They found that PARP-6 is expressed at high levels throughout neuronal development, specifically during dendrite morphogenesis. PARP-6 knockdown in primary rat hippocampal neurons resulted in decreased dendritic complexity. Ectopic expression of wild-type, but not catalytically dead, PARP-6 increased dendritic complexity, demonstrating a requirement for PARP-6 catalytic activity [76]. Although the targets of PARP-6 MARylation in the neurons have not been identified, this study nicely demonstrates a role for the cytosolic MART PARP-6 and its catalytic activity during neuronal development.

Like PARP-6, PARP-7 is also highly expressed in the brain [77]. Using a *Parp7* (a.k.a. *Tiparp*) knockout mouse, Grimaldi et al. (2019) discovered a role for PARP-7 in the development of the prefrontal cortex [78]. Abnormal organization of the cortex was observed in these mice, with higher cell density in the upper layers. Genetic depletion of PARP-7 disrupted both the distribution and number of GABAergic neurons, as well as a reduction in neural progenitor cell proliferation and migration [78]. Interestingly, the levels of α-tubulin MARylation were reduced in *Parp7* knockout cells, which may be related to the PARP-7-dependent increase in microtubule instability described above [33].

### 4.2. PARP-7 and Responses to Environmental Toxins

Dioxin (2,3,7,8-tetrachlorodibenzo-p-dioxin; TCDD) is a highly toxic environmental contaminant that can cause a range of toxic responses, including hepatic damage, steatohepatitis, and lethal wasting syndrome [79]. Dioxin exposure has also been linked to appetite suppression, reduced body weight, and suppression of energy homeostasis [79]. Toxic effects of dioxin are mediated by the aryl hydrocarbon receptor (AHR), a cytosolic protein that re-localizes to the nucleus after binding dioxin [80]. There, it functions as a sequence-specific DNA binding transcription factor to regulate the transcription of target genes, including drug metabolizing enzymes [80]. The gene encoding PARP-7, also known as TCDD-inducible poly(ADP-ribose) polymerase (TiPARP), is one target of AHR.

Treatment of *Parp7* knockout mice with dioxin revealed some interesting differences versus wild-type mice [81]. Wild-type mice survived more than 30 days after a single 100 mg/kg dose of dioxin, while no *Parp7* knockout mice injected with dioxin survived past 5 days. Genetic depletion of PARP-7 increased the incidence of steatohepatitis after dioxin injection. Mechanistically, PARP-7 catalytic activity is required for the MARylation of AHR, but not its binding partner, the aryl hydrocarbon receptor nuclear translocator (ARNT) [81]. Collectively, these studies have revealed a role for PARP-7 and its catalytic activity in AHR-mediated responses to dioxin.

### 4.3. PARP-7 in Stem Cells and Cancer

In addition to its role in AHR-mediated responses to dioxin, PARP-7 has been implicated in the biology of embryonic stem cells and cancers. PARP-7 was identified as a factor that safeguards pluripotency in embryonic stem cells (ESCs) by protecting key pluripotency genes (e.g., *Nanog*, *Pou5f1*, *Sox2*, *Stella*, *Tet1*, and *Zfp42*) from progressive epigenetic repression [82]. In the absence of PARP-7, ESCs exhibit a decrease in ground state pluripotency and a higher propensity to differentiate. Thus, PARP-7 plays a role in determining the developmental plasticity of ESCs [82].

Furthermore, as noted above, PARP-7 has been found to play a key role in cancers, acting to promote the growth of ovarian and other cancer cell types [33]. Some evidence suggests that gene amplifications drive elevated *PARP7* mRNA expression in malignant cells in ovarian cancers, although this may not be the case in other cancer types (e.g., breast). In this regard, *PARP7* mRNA expression is lower in tumor tissues compared to normal tissues, and higher *PARP7* mRNA is associated with better survival outcomes [83,84]. Knockdown of *PARP7* in ovarian and kidney cancer cells leads to reduced proliferation [33], whereas knockdown of *PARP7* in breast and colon cancer xenografts promotes enhanced tumor formation [84]. Thus, there may be context-dependent functions of PARP-7.

Mechanistically, PARP-7 may affect tumor formation and survival by modulating (1) microtubule stability by MARylation of ⍺-tubulin, as noted above [33], and (2) modulating cancer-directed host immune responses [31]. The ability to quickly depolymerize and disassemble microtubules may be important for efficient cell proliferation and migration. Note in this regard that taxanes and related molecules function as anticancer drugs that stabilize microtubules, leading to the arrest of proliferation and mitosis [85]. Moreover, the ability of cancer to evade cancer-directed host immune responses can be an important mechanism for continued cancer growth.

### 4.4. PARP-14 in Immune Cell Regulation

In 2005, PARP-14 was identified as a member of the B-aggressive-lymphoma (BAL) protein family [86]. The following year, Goenka and Boothby found that PARP-14 collaborates with STAT6 to modulate IL-4-induced gene expression [87]. They also found that PARP-14 catalytic activity can enhance Stat6 transcriptional responses [88]. More recently, Cho et al. (2011) observed a role for PARP-14 in the STAT6-dependent processes required for T-cell and B-cell responses [89]. They determined that PARP-14 is required for both IL-4-regulated glycolysis and glucose oxidation in T-cells and B-cells, as well as IL-4-induced pro-survival signaling in B-cells [89].

Subsequent studies have shown that Th2 cells isolated from *Parp14* knockout mice exhibit decreased production of Th2-specific cytokines, including IL-4, IL-5 and IL-13 [90]. A proallergenic role of PARP-14 was identified in induced allergic airway disease (AAD) using *Parp14* knockout mice. In this regard, the knockout animals exhibited decreased immune cell infiltration into the lungs, together with decreased IL-4, IL-5, and IL-13 expression [90]. In an effort to elucidate the role of PARP-14 catalytic activity in the immune response, the mice were treated with PJ34, a broad-specificity PARP inhibitor that can inhibit PARP-14. Treatment with PJ34 reduced AAD in mice [90]. Because PJ34 is not a PARP-14-specific inhibitor, the authors treated *Parp14* knockout mice with the drug and saw no further decrease, supporting the conclusion that, in this model, the catalytic inhibition by PJ34 is specific to PARP-14. Together, these studies demonstrate a role for PARP-14 in immune cell regulation, an aspect of biology that is impacted by many members of the PARP family.

## 5. Development of MART Inhibitors

In the past decade, PARP inhibitors have made their way from the lab to the clinic, with demonstrated therapeutic benefits [91]. So far, clinical benefit of PARP inhibitors in cancer treatment has been limited to inhibitors of nuclear PARPs (e.g., PARP-1) [91]. As discussed above, MARTs are exciting therapeutic targets for several diseases, but the development of clinical inhibitors of these MARTs has lagged behind. However, recent advances in the understanding of MART structure and catalytic activity, as well as the ability to detect MARylation, has led to significant progress in developing MART inhibitors [4,91]. In many cases, auto-modification of the MART is used as readout for catalytic activity. In this section, we summarize recent advances that have enabled discovery of inhibitors of MARTs, listed in PARP numerical order.

### 5.1. PARP-7 Inhibitors

**Figure 5:**
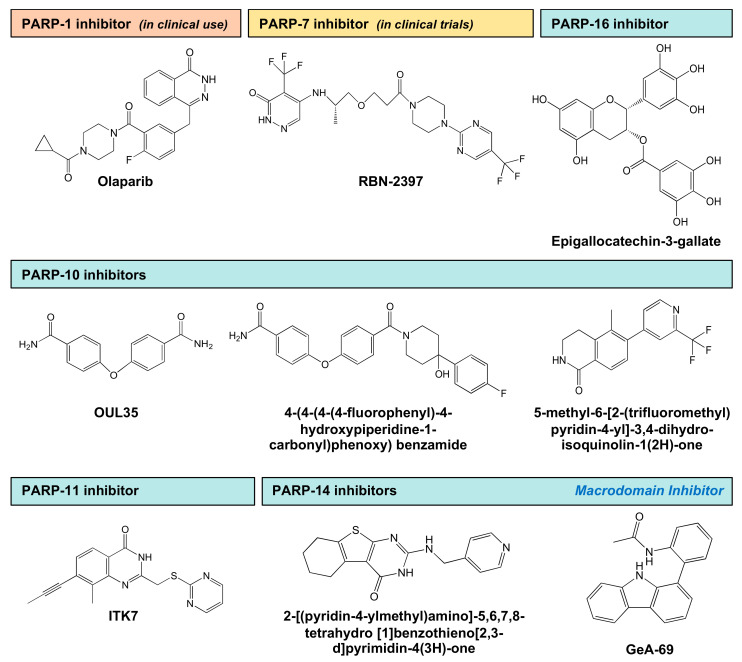
Chemical structures of PARP and MART inhibitors. The structure of the PARP-1 inhibitor, Olaparib, the first FDA-approved PARP inhibitor, is shown for comparison. The PARP-7 inhibitor, RBN-2397, is in Phase I clinical trials (ClinicalTrials.gov identifier: NCT04053673). All of the inhibitors shown target the catalytic domain of the PARPs, except the PARP-14 inhibitor GeA-69, which targets the macrodomains. See also Table 2.

As noted above, PARP-7 plays key roles in aspects of biology that can impact human health, including responses to environmental toxins, neuronal function, and cancer. As such, it is an attractive target for potential therapeutics. Ribon Therapeutics, Inc. has developed a potent and selective PARP-7 inhibitor (RBN-2397), which is now in Phase I clinical trials for solid tumors (ClinicalTrials.gov identifier: NCT04053673) (Figure 5, Table 2). RBN-2397 is a first-in-class inhibitor of a MART, representing a previously unexplored class of therapeutic targets. It was developed using a platform of high-throughput biochemical and cellular MART assays, and a family-wide screening panel [92,93,94]. Cancer cells use PARP-7 to suppress the type I interferon (IFN) response to cytosolic nucleic acids. RBN-2397 inhibits PARP-7 to restore type I IFN signaling in the tumor, causing complete tumor regression and adaptive immunity in mouse models [92]. In this review, we have highlighted roles for PARP-7 in a variety of biological processes, including potential tumor suppressor activity in breast cancers and tumor promoting activities in ovarian cancers. The diverse biological roles for PARP-7 suggest that therapeutic inhibition will be disease context-dependent.

### 5.2. PARP-10 Inhibitors

PARP-10 is an attractive therapeutic target in cancer, since it regulates cell proliferation through multiple mechanisms, including the regulation of ß-catenin, and relieving replication and oxidative stress [95,96,97]. Putt et al. (2004) have described a fluorescent assay to measure the levels of PARP-10 activation by quantifying the levels of NAD^+^ consumption [98]. In this assay, the authors used the unique property of *N*-alkylpyridinium to convert to fluorescent molecules after reaction with ketones and acid at high temperatures [98]. Venkannagari et al. (2013) used this assay to screen a library of natural compounds to identify inhibitors of PARP-10 and PARP-15 [98]. They identified naphthoquinones, psoralens, and flavones as potential inhibitors of PARP-10, although all have low potency. In a follow-up study, the screen was performed across a larger library of chemical compounds, resulting in the identification of OUL35 as an inhibitor of PARP-10 catalytic activity [99] (Figure 5, Table 2). OUL35 treatment rescues PARP-10-induced cell death and sensitizes cells to hydroxyurea [99]. Ekblad et al. (2015) used a structure-guided medicinal chemistry approach to identify potential inhibitors of PARPs-10, 14 and 15. These compounds require interactions with the nicotinamide pocket of the MART catalytic domain for complete inhibition of enzyme activity [100]. Holechek et al. (2018) modified OUL35 to identify PARP-10/PARP-14 selective inhibitors that have good solubility and metabolic stability against murine liver microsomes [101].

Morgan et al. (2015) used a chemical genetics approach for generating selective inhibitors of a mutant of PARP10 (LG-PARP10) that contains a unique pocket in its active site [102]. They generated a series of C-7 substituted 3,4-dihydroisoquinolin-1(2H)-one (dq) analogues designed to selectively inhibit LG-PARP10 [102] (Figure 5, Table 2). In follow up studies, they used structure-based design to target a hydrophobic sub-pocket within the nicotinamide-binding site of PARP-10 [103]. In this case, they used a series of small molecules based on the dq scaffold contain substituents at the C-5 and C-6 positions designed to exploit the hydrophobic sub-pocket. They identified an analogue that contains a methyl group at the C-5 position and a substituted pyridine at the C-6 position, which exhibits >10-fold selectivity for PARP-10 over many other PARP family members [103].

### 5.3. PARP-11 Inhibitors

Kirby et al. (2018) have developed specific inhibitors of PARP-11 by taking advantage of structural differences between the active sites of PARPs that mediate MARylation versus PARylation [104]. The glutamate residue in the H-Y-E active site triad in PARP polyenzymes is replaced by hydrophobic amino acids in MARTs. Using a quinazolin-4(3H)-one scaffold (QDR scaffold) from a pan-PARP inhibitor that binds to the nicotinamide site of PARP-14, the authors synthesized various derivatives and found that (1) substitution at the C-7 position increased the selectivity against PARP polyenzymes and (2) substitution at the C-2 position improved specificity within the family of PARP monoenzymes. Addition of a propynyl modification at the C-7 position and p-benzoic acid at the C-2 position (e.g., ITK6) increased the potency towards PARP-10 and PARP-11. Similarly, the addition of propynyl at the C-7 and pyrimidine at the C-2 position (e.g., ITK7) enhanced the potency against PARP-11. [104]. As previously described, PARP-11 localizes to the nuclear pore and modifies key proteins involved in the organization of nuclear pores [2]. Interestingly, treatment with ITK7, a selective PARP-11 inhibitor (Figure 5, Table 2), abrogated nuclear pore localization of PARP-11 [104]. These results demonstrate the value of selective inhibitors in discovering and exploring new biology.

### 5.4. PARP-14 Inhibitors

PARP-14 plays an important role in inflammation and cancer, making it an important therapeutic target. PARP-14 contains a PARP catalytic domain, as well as three macrodomains that bind ADPR (Figure 1). Efforts to develop small molecule PARP-14 inhibitors have focused on inhibition of the catalytic activity or by binding to the macrodomains to inhibit their function. Yoneyama-Hirozane et al. (2017) used high-throughput mass spectrometry (HTMS) and an immunoradiometric assay with [^3^H]NAD^+^ to identify hit compounds [105]. The HTMS method was used to quantify the levels of nicotinamide produced as a product of NAD^+^ consumption by PARP-14. The compounds identified bind to PARP-14 at the NAD^+^ binding site and, hence, inhibit PARP-14 activity by competing for binding with NAD^+^ (Figure 5, Table 2). In addition, treatment with these inhibitors resulted in increased stability of PARP-14 protein [105].

Since the catalytic domains of the various PARP family members are structurally similar (Figure 1), the development of PARP-selective inhibitors can be a challenge. Thus, targeting of other unique structural features in PARPs, such as macrodomains, may provide an alternate approach for developing inhibitors. AlphaScreen (amplified luminescent proximity homogenous assay screen) technology is a useful approach for high throughput screening to quantify analyte accumulation or depletion, bimolecular interactions, and post-translational modifications [109]. It has been used in a format in which blocking of a macrodomain with a small molecule leads to displacement of “donor” beads from “acceptor” beads whose proximity is mediated by binding of the macrodomain with ADPR [107,110]. This assay programmed with a MARylated and biotinylated peptide, and recombinant macrodomain repeats, was used to identify GeA-69, a kinase inhibitor, as an inhibitor of PARP-14 macrodomain 2 [107,110] (Figure 5, Table 2). GeA-69 is selective for PARP-14 macrodomain 2, cell permeable, and effective at preventing the localization of macrodomain 2 to site of DNA damage in intact cells [107,110]. These results demonstrate the potential utility of targeting other functional domains in PARPs.

### 5.5. PARP-16 Inhibitors

PARP-16 localizes to the endoplasmic reticulum and modifies mediators of ER stress responses, such as PERK and IRE1α [58]. Moreover, PARP-16 regulates Sec-body formation in *Drosophila* in response to amino acid starvation (Sec-bodies are reversible, non-membrane bound stress assemblies that incorporate components of ER exit sites) [43]. These data suggest that PARP-16 plays an essential role in stress responses, making it an important therapeutic target for cancer. One study has identified epigallocatechin-3-gallate (EGCG), a component of green tea, as an inhibitor of PARP-16 activity, which antagonizes the UPR and promotes ER stress-induced cell death [108] (Figure 5, Table 2). Importantly, this compound has very low potency, hence a true inhibitor for PARP-16 has yet to be developed. Wigle et al. (2020) have developed novel chemical probes and TR-FRET (time-resolved fluorescence resonance energy transfer) and NanoBRET (NanoLuc-based bioluminescence resonance energy transfer) assays to characterize the inhibitors of PARP-16 that compete for NAD^+^ binding [94]. These FRET and BRET probes were generated using RBN010860, a pan-MART inhibitor that binds to H-Y-I/L/Y PARPs, including PARP-16 [94].

### 5.6. Conclusions: MART Inhibitors

The studies highlighted here have generated a wealth of resources for MART inhibitor development, including high throughput assays, structural information, and chemical matter. Although the efficacy of these inhibitors in vivo and in patients are yet to be evaluated, the available inhibitors are invaluable tools that can provide important information for developing more potent and selective inhibitors. Clinically efficacious inhibitors of MARTs will be useful for treating diseases such as cancer, inflammation, and viral infections.

## 6. Future Perspectives

MARTs are a biologically and therapeutically important class of enzymes that regulate diverse cellular pathways and play an essential role in several diseases. Recent technological advances have enabled better detection of MARylation and chemical inhibition of MART catalytic activity. But, many of the biological functions and regulatory pathways of MARylation have yet to be elucidated. Key aspects requiring further understanding are (1) the in vivo “readers” of MARylation, (i.e., the proteins that bind MAR to elicit functional outcomes) and (2) the “erasers” of MARylation (i.e., the ADPR hydrolases that remove MAR from proteins). In addition, a greater emphasis on identification and functional assessment of specific sites of MARylation on key substrate proteins is required to fully understand the biological consequences of these modifications.

## 7. Patents

U.S. Patent 9,599,606 covers the set of ADP-ribose detection reagents described herein, which have been licensed to and are sold by EMD Millipore. U.S. Patent 9,926,340 covers the clickable NAD^+^ analogs and analog sensitive PARP mutants that allow PARP-specific ADP-ribosylation of proteins described herein.

## Figures and Tables

**Table 1:** Summary of MART enzymes.

MART Enzyme ^1^	Catalytic Triad Sequence	Localization ^2^	Key Functions ^3^
**PARP-3**	H-Y-E	Nuclear	DNA damage repair
**PARP-4**	H-Y-E	Cytosolic (Vault particles)	Vault particle function
**PARP-6**	H-Y-I	Cytosolic	Dendrite complexity
**PARP-7**	H-Y-I	Nuclear/Cytosolic	Viral response, Gene regulation Cytoskeleton regulation
**PARP-8**	H-Y-I	Cytosolic	Cell viability
**PARP-10**	H-Y-I	Cytosolic	DNA damage repair
**PARP-11**	H-Y-I	Cytosolic	Nuclear pore function
**PARP-12**	H-Y-I	Cytosolic	Stress granule function
**PARP-14**	H-Y-L	Cytosolic	Cytoskeleton regulation, Immune response
**PARP-15**	H-Y-L	Cytosolic	Stress granule function
**PARP-16**	H-Y-Y	Cytosolic (Endoplasmic reticulum)	ER stress responses

^1^ This list includes all PARP family members with MART activity, regardless of their subcellular localization. ^2^ Based primarily on data from Vyas et al., 2014 [2]. ^3^ Selected functions based on the literature. Not comprehensive.

**Table 2:** Summary of MART inhibitors.

PARP	Inhibitor	Mode of Inhibition ^1^	Efficacy ^2^	Reference
**PARP-7**	RBN-2397	NAD^+^ binding pocket	50% inhibition at 3 nM	[106]
**PARP-10**	Naphthoquinones	Unknown	55–67% inhibition at 10 µM	[98]
	Psoralens	Unknown	61–69% inhibition at 10 µM	[98]
	Flavones	Unknown	78% inhibition at 10 µM	[98]
	OUL35	NAD^+^ binding pocket	50% inhibition at 239 nM	[99]
	*N*^1^-(3-carbamoylphenyl)-*N*^4^-methylmaleamide	NAD^+^ binding pocket	50% inhibition at 2 µM	[100]
	4-(4-(4-(4-fluorophenyl)-4-hydroxypiperidine-1-carbonyl)phenoxy) benzamide	NAD^+^ binding pocket	50% inhibition at 0.39 µM	[101]
	5-methyl-6-(2-(trifluoromethyl)pyridin-4-yl)-3,4-dihydroisoquinolin-1(2H)-one	NAD^+^ binding pocket	50% inhibition at 1.8 µM	[103]
**PARP-11**	ITK7	Hydrophobic sub-pocket of H-Y-Φ	50% inhibition at 14 nM	[104]
**PARP-14**	6-{3-[4-(diphenylmethoxy)piperidin-1-yl]propoxy} [1,2,4]triazolo[4,3-b]pyridazine	NAD^+^ binding pocket	50% inhibition at 0.58 µM	[105]
	2-[(pyridin-4-ylmethyl)amino]-5,6,7,8-tetrahydro [1]benzothieno[2,3-d]pyrimidin-4(3H)-one	NAD^+^ binding pocket	50% inhibition at 0.31 µM	[105]
	GeA-69	Macrodomain	50% inhibition at 0.72 µM	[107]
**PARP-16**	Epigallocatechin-3-gallate	Unknown	50% inhibition at 14.52 µM	[108]

^1^ Mode of inhibition: The specified compound binds to; (1) the “NAD+ binding pocket” of the PARP enzyme; (2) the “hydrophobic sub-pocket of H-Y-Φ” in the PARP enzyme; (3) a “macrodomain” in the PARP enzyme; or is “unknown.” ^2^ Percent inhibition values are rounded to the nearest 10 and are listed as the maximum inhibition achieved at the indicated concentration. The concentrations listed are rounded to one or two significant digits. See Figure 5 for the chemical structures of these compounds.
